# Paradoxical markers of conscious levels: Effects of propofol on patients in disorders of consciousness

**DOI:** 10.3389/fnhum.2022.992649

**Published:** 2022-10-06

**Authors:** Charlotte Maschke, Catherine Duclos, Stefanie Blain-Moraes

**Affiliations:** ^1^Montreal General Hospital, McGill University Health Centre, Montreal, QC, Canada; ^2^Integrated Program in Neuroscience, McGill University, Montreal, QC, Canada; ^3^Hôpital du Sacré-Cœur de Montréal, Centre Intégré Universitaire de Santé et de Services Sociaux du Nord-de-l’île-de-Montréal, Montreal, QC, Canada; ^4^Department of Anesthesiology and Pain Medicine, Université de Montréal, Montreal, QC, Canada; ^5^School of Physical and Occupational Therapy, McGill University, Montreal, QC, Canada

**Keywords:** disorders of consciousness, anesthesia, functional connectivity, complexity, electroencephalography

## Abstract

Human consciousness is widely understood to be underpinned by rich and diverse functional networks, whose breakdown results in unconsciousness. Candidate neural correlates of anesthetic-induced unconsciousness include: (1) disrupted frontoparietal functional connectivity; (2) disrupted brain network hubs; and (3) reduced spatiotemporal complexity. However, emerging counterexamples have revealed that these markers may appear outside of the state they are associated with, challenging both their inclusion as markers of conscious level, and the theories of consciousness that rely on their evidence. In this study, we present a case series of three individuals in disorders of consciousness (DOC) who exhibit paradoxical brain responses to exposure to anesthesia. High-density electroencephalographic data were recorded from three patients with unresponsive wakefulness syndrome (UWS) while they underwent a protocol of propofol anesthesia with a targeted effect site concentration of 2 μg/ml. Network hubs and directionality of functional connectivity in the alpha frequency band (8–13 Hz), were estimated using the weighted phase lag index (wPLI) and directed phase lag index (dPLI). The spatiotemporal signal complexity was estimated using three types of Lempel-Ziv complexity (LZC). Our results illustrate that exposure to propofol anesthesia can paradoxically result in: (1) increased frontoparietal feedback-dominant connectivity; (2) posterior network hubs; and (3) increased spatiotemporal complexity. The case examples presented in this paper challenge the role of functional connectivity and spatiotemporal complexity in theories of consciousness and for the clinical evaluation of levels of human consciousness.

## Introduction

The quest for a biological understanding of consciousness has emerged as one of the most fundamental scientific pursuits of the 21st century. Many prominent neuroscientific theories of consciousness posit that organized integration of cortical areas is central to the conscious state (Tononi et al., 2016; Carhart-Harris, 2018; Mashour et al., 2020). In empirical studies of sleep, general anesthesia and disorders of consciousness (DOC), these theories have successfully predicted that a breakdown in either the strength or the repertoire of functional brain connections accompanies unconsciousness (Massimini et al., 2005; Casali et al., 2013; Jordan et al., 2013; Lee H. et al., 2013; Blain-Moraes et al., 2014; Barttfeld et al., 2015; Chennu et al., 2016; Ranft et al., 2016). Specifically, studies have suggested that (1) disrupted frontoparietal functional connectivity (Ku et al., 2011; Boly et al., 2012; Jordan et al., 2013; Bonhomme et al., 2016; Pal et al., 2016; Sanders et al., 2018); (2) disrupted brain network hubs (Achard et al., 2012; Schröter et al., 2012; Lee H. et al., 2013; Moon et al., 2015); and (3) reduced spatiotemporal complexity, correlated with reduced levels of consciousness (Sitt et al., 2014; Schartner et al., 2015, 2017; Hudetz et al., 2016; Wang et al., 2017; Frohlich et al., 2020). Disruption of frontoparietal functional connectivity and brain network hubs is predominantly expressed in the alpha frequency band (8–13 Hz) (Lee H. et al., 2013; Blain-Moraes et al., 2014, 2017; Kallionpää et al., 2020). Although these measures of brain network function have been proposed as potential neural correlates of consciousness (Crick and Koch, 2003), emerging counterexamples have revealed that they may paradoxically appear outside of the state they are associated with. For example, a recent seminal study demonstrated that these connectivity measures could be dissociated from states of wakefulness and unconsciousness in rats receiving cholinergic stimulation to the prefrontal cortex while exposed to anesthesia (Pal et al., 2020). Such paradoxical dissociations are useful both for selecting amongst candidate neural correlates of consciousness, and also for evaluating different theories of consciousness by falsifying those that are incompatible with the observed dissociation.

In a recent study (Duclos et al., 2022) we presented the Adaptive Reconfiguration Index (ARI), which measures the amount of reconfiguration of directed functional connectivity and node degree in brain networks constructed from the electroencephalogram (EEG) of individuals exposed to a targeted dose of propofol anesthesia. In this pilot study with individuals in DOC, we demonstrated that the ARI could predict whether or not a patient would recover responsiveness within 3 months. The ARI is grounded in the expectation that directed functional connectivity and network hub location are markers of level of consciousness. Regardless of the state of the pathologically altered brain network at baseline, we expected that exposure to propofol would be accompanied by markers of reduced consciousness (i.e., neutralization of feedback-dominant frontoparietal connectivity and anteriorization of brain network hubs) in patients with the capacity to recover consciousness. The rationale underpinning the ARI parallels that of the Perturbational Complexity Index (PCI) (Casali et al., 2013)–a popular measure of consciousness level based upon the Lempel-Ziv Complexity (LZC) of brain networks perturbed by transcranial magnetic stimulation. Interestingly, in our previous study (Duclos et al., 2022), we demonstrated that the amount of brain network reconfiguration induced by anesthetic perturbation was predictive of capacity for consciousness, independently of the expected direction of reconfiguration. Although some of the participants who recovered consciousness showed brain network reconfiguration consistent with expected markers of reduced consciousness, several participants showed paradoxical reconfigurations contrary to these expectations.

In the current study, we present a case series of three examples of the paradoxical reconfigurations collected as part of these experiments (Duclos et al., 2022) and demonstrate that (1) frontoparietal feedback-dominant connectivity can increase under anesthesia and (2) network hubs can become posterior-dominant during exposure to anesthesia. Concurrently, we calculate the LZC of the brain networks in each case and demonstrate that and (3) spatiotemporal complexity can increase during exposure to anesthesia. These findings challenge the role of measures of network connectivity and complexity in theories of consciousness for the clinical monitoring of consciousness.

## Materials and methods

### Participants

Three individuals in a DOC were included in this case series. Individuals in a DOC were included following acquired brain injury (anoxic, traumatic, hypoxic brain injury, stroke). Patients were assessed by a trained experimenter using the Coma Recovery Scale-Revised (CRS-R) (Kalmar and Giacino, 2005) on the day of the study. All three patients were diagnosed as having an Unresponsive Wakefulness Syndrome (UWS). Patients were excluded if they were diagnosed with status epilepticus or receiving sedation at the time of the study. For all participants, written informed consent was provided by their legal representative in accordance with the Declaration of Helsinki. The study was approved by the McGill University Health Center Research Ethics Board (15-996-MP-CUSM). Acute patients’ clinical outcomes were assessed 3 months post-EEG through their capacity to follow commands.

### Electroencephalography data

Electroencephalogram data were recorded from a 128-channel EGI Sensor Net using an Amps 400 amplifier (Electrical Geodesic, Inc., USA), a sampling rate of 1 kHz and vertex reference. Electrode impedance was reduced to below 5 kΩ prior to data collection, as per manufacturer recommendation. Prior to functional connectivity analysis, the raw signal was average referenced and downsampled to 250 Hz. The data were high pass filtered at 0.1 Hz, low-pass filtered at 45 Hz and notch filtered at 60 Hz. Channels with an excessive level of noise were removed prior to average referencing. The signal was epoched into 10 s windows of continuous data and visually inspected by a trained investigator to manually reject signal sections containing non-physiological artifacts. Non-brain channels were removed from the subsequent analysis.

### Anesthetic protocol

All three participants in a DOC underwent an anesthetic protocol, described in our previous work (Blain-Moraes et al., 2016; Duclos et al., 2022). The first EEG recording was performed before the start of the anesthetic protocol (herein referred to as Baseline state). Subsequently, Propofol was administered in a target-controlled infusion pump. Once the effect size concentration of 2 μg/ml was reached, the concentration was held constant. A third recording was performed as propofol reached a concentration lower than 0.5 μg/ml (herein referred to as post-anesthesia). For the purpose of this study, we analyzed 5 min of baseline EEG and 5 min of EEG during constant concentration of propofol. A comparison between the Baseline state and the post-anesthesia state is provided in the Supplementary material.

### Functional connectivity analysis

We bandpass filtered the signal at the alpha bandwidth (8–13 Hz), as evidence from previous research has implicated this frequency band as providing discriminatory information in consciousness and awareness (Lee H. et al., 2013; Blain-Moraes et al., 2014, 2017; Kallionpää et al., 2020). Weighted and directed functional connectivity were estimated using the weighted phase lag index (wPLI) and directed phase lag index (dPLI) using custom python functions, available at https://github.com/BIAPT/Python_Connectivity.

#### Functional connectivity strength and network hubs

The strength of functional connectivity was estimated using the wPLI, a phase-based measure of functional connectivity (Vinck et al., 2011). The wPLI is defined by the phase difference between two signals *s_i_* and *s_j_*, weighted by the magnitude of the imaginary component of the cross-spectrum *𝒥*(*C*_*ij*_):


$$w\,P\,L\,I_{i\,j}=\frac{\left|E\,\left\{𝒥\,\left(C_{i\,j}\right)\right\}\right|}{E\,\left\{\left|𝒥\,\left(C_{i\,j}\right)\right|\right\}}=\frac{\left|E\,\left\{\left|𝒥\,\left(C_{i\,j}\right)\right|\,s\,g\,n\,\left(𝒥\,\left(C_{i\,j}\right)\right)\right\}\right|}{E\,\left\{\left|𝒥\,\left(C_{i\,j}\right)\right|\right\}}$$

where E{.} denotes the expected value operator and sgn(.) refers to the sign function (Vinck et al., 2011). The wPLI has values of 0 ≤ wPLI ≤ 1, with 1 indicating a strong functional coupling relationship and 0 indicating no functional connectivity. The wPLI was calculated using a non-overlapping sliding window approach with a window- and step size of 10 s and averaged over time subsequently. Network hubs were estimated using node degree (i.e., summed connection from one electrode to all other electrodes) of the time-averaged wPLI matrix.

#### Directed functional connectivity

Directed functional connectivity was estimated using the dPLI, a phase-based measure of functional connectivity that estimates the phase lead-lag relationship between two signals *s_i_* and *s_j_* (Stam and van Straaten, 2012). The phase angle difference △*ϕ*_*ij*_ was computed with a Hilbert transform. The dPLI was then calculated using the following formula:


$$d\,P\,L\,I_{i\,j}=\frac{1}{N}\,\sum_{t=1}^{N}H\,\left(△\,\phi_{i\,j}\right)$$

where *N* denotes the length of the analysis segment, *t* the given time point and *H* the Heaviside step function (Stam and van Straaten, 2012). A dPLI value of 0 < dPLI_*ij*_ ≤ 0.5 indicates *s_i_* phase-lagging *s_j_*, values of 0.5 < dPLI_*ij*_ ≤ 1 express *s_i_* phase-leading *s_j_*. A dPLI of 0.5 occurs if there is no phase relationship between *s_i_* and *s_j_*. The dPLI was calculated using a non-overlapping sliding window approach with a window- and step size of 10 s and averaged over time subsequently. Frontoparietal connectivity was estimated by filtering connections between frontal and parietal regions within each hemisphere separately.

#### Surrogate analysis

Both estimates of functional connectivity were controlled for spurious connectivity by performing a surrogate data analysis. Twenty surrogate datasets were generated for each combination of channel pairs *i* and *j* by maintaining the time series of channel *i* and randomly scrambling the time series of channel *j*. The wPLI and dPLI values of each time step were corrected by the mean of the corresponding surrogate set. The original values were retained if they were significantly different from the surrogate dataset’s distribution (*p* < 0.05). Non-significant connections were set to 0 for wPLI and 0.5 for dPLI.

### Signal complexity

Signal complexity was calculated using LZC, which is an estimate of the compressibility and information-richness of a signal (Lempel and Ziv, 1976). Using the code provided by Toker et al. (2022), three different types of LZC were calculated for this study. For all three types of LZC, the signal was binarized using the mean of the signal’s instantaneous amplitude (Schartner et al., 2015). We then computed (1) the median univariate LZC, (2) the joint LZC, and (3) the concatenated LZC. For the median univariate LZC, the complexity of every channel was estimated individually prior to averaging. For the concatenated LZC, data of all channels were concatenated into a single array as proposed by Schartner et al. (2015). Whereas the univariate and concatenate LZC both estimate complexity based on the compression of a one dimensional time series, the joint LZC uses a multivariate compression algorithm which allows to calculate complexity over multivariate spatiotemporal data directly (Zozor et al., 2005). All types of LZC were calculated on non-overlapping 10 s windows of EEG, and then averaged to produce an overall measure of complexity for each state (Baseline and Anesthesia). To minimize the influence of spectral changes on the complexity estimate, LZC was normalized with phase-randomized surrogates, as described in Toker et al. (2022).

## Results

### Case 1

A 38-year-old female was admitted to the intensive care unit due to severe headache. The computer tomography scan presented severe intraventricular hemorrhage in lateral third and fourth ventricles with ventriculomegaly and hydrocephalus due to a ruptured right frontal periventricular arteriovenous malformation. Data acquisition was conducted 23 days post-injury, when the patient scored 5 on the Glasgow Coma Scale. At this time, the patient did not present new intra or extra-axial hemorrhage and did not show any significant interval change in the size and configuration of the supratentorial ventricular system. At the day of recording, the patient was classified to be in an UWS, determined by a CRS-R score of 5 (Table 1). Within 3 months post-EEG the patient recovered consciousness and was able to consistently follow commands. Due to a small bandage in the left frontal region, 10 channels were removed in addition to non-brain electrodes prior to analysis (Supplementary Figure 1).

**TABLE 1:** Demographic and clinical data of patients.

Case	Age	Sex	Etiology	State	Diagnose	CRS-R score						
						
						Auditory	Visual	Motor	Oromotor	Communication	Arousal	Total
1	38	F	ICH	Acute	UWS	1	0	2	0	0	2	5
2	75	F	Stroke	Acute	UWS	1	1	2	0	0	1	5
3	28	F	Anoxic	Chronic	UWS	1	0	1	2	0	2	6

**FIGURE 1:**
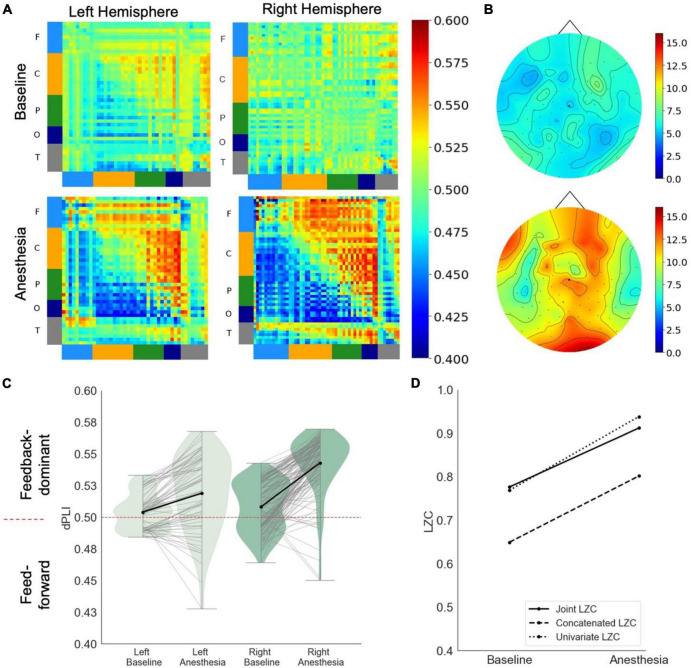
Case 1 **(A)** directed functional connectivity (dPLI) within the right and left hemisphere during baseline and anesthesia. Electrodes are ordered per region: F, frontal; C, central; P, parietal; T, temporal; O, occipital **(B)** network hubs during baseline and anesthesia. **(C)** Directed functional connectivity (dPLI) between frontal and parietal regions within the left and right hemisphere. Light gray lines represent individual electrodes, black lines represent the mean. **(D)** Three types of Lempel-Ziv complexity and its propofol-induced change.

In response to propofol anesthesia, this participant exhibited a strong increase in global feedback-dominant connectivity in both hemispheres (Figure 1A), as shown by anterior regions phase-leading more posterior regions of the brain (i.e., dPLI larger than 0.5). Focusing solely on connections between frontal and parietal regions revealed an increase in feedback-dominance in the left and right hemisphere (Figure 1C). The network node degree globally increased in response to propofol, with the strongest hub being centered in the back of the brain under anesthesia (Figure 1B). The joint, univariate and concatenated LZC increased under propofol anesthesia (Figure 1D). During the post-anesthetic state, the anesthetic-induced changes in functional connectivity, network hubs and complexity were reversed and reapproached the Baseline level (see Supplementary Figure 2).

### Case 2

A 75-year-old female was admitted to the intensive care unit due to severe headache. The computer tomography scan revealed a diffuse subarachnoid hemorrhage with predominant infratentorial distribution and intraventricular extension and hydrocephalus [Fisher Grade IV, World Federation of Neurosurgeons (WFNS) Grade V] due to a ruptured left posterior inferior cerebellar artery aneurysm. She also presented a mild right frontal subdural pneumocephalus and an unruptured saccular aneurysm of the cavernous segment of the left internal carotid artery. She suffered a right cerebellar infarct 5 days after admission. In addition to the brain injury, the patient suffered from hypertension and dyslipidemia. Data acquisition was conducted 10 days post-injury. The patient was classified to be in an UWS, determined by a CRS-R score of 5 (Table 1). The patient died within 1 month post-injury. At this time, the patient had not recovered full consciousness.

Due to a large bandage in the right frontocentral region, 24 channels were removed in addition to non-brain electrodes prior to analysis (see Supplementary Figure 1). Due to the weak electrode coverage of the right hemisphere, we focus our interpretation on the left hemisphere.

**FIGURE 2:**
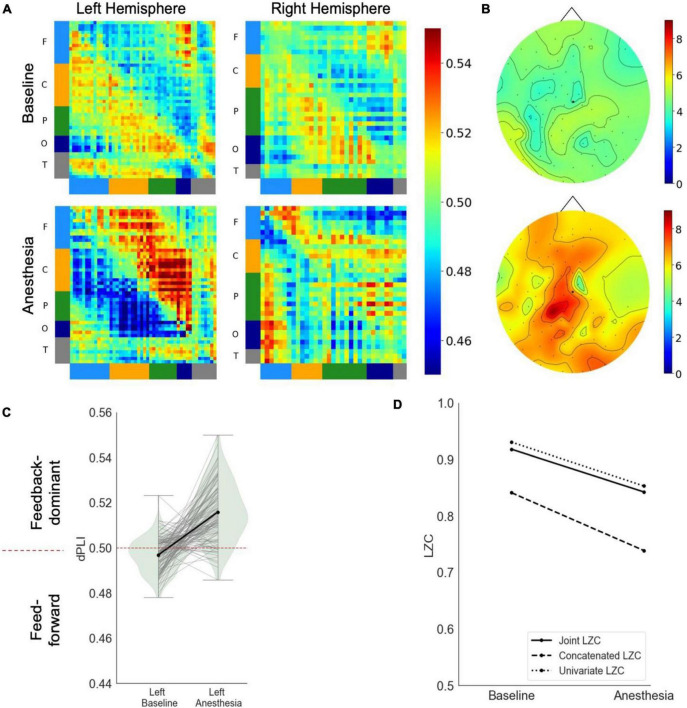
Case 2 **(A)** directed functional connectivity (dPLI) within the right and left hemisphere during baseline and anesthesia. Electrodes are ordered per region: F, frontal; C, central; P, parietal; T, temporal; O, occipital **(B)** network hubs during baseline and anesthesia. **(C)** Directed functional connectivity (dPLI) between frontal and parietal regions within the left and right hemisphere. Light gray lines represent individual electrodes, black lines represent the mean. **(D)** Three types of Lempel-Ziv complexity and its propofol-induced change.

In response to propofol anesthesia, this participant exhibited a strong increase in global feedback-dominant connectivity in the left hemisphere (Figure 2A). Focusing solely on connections between frontal and parietal regions revealed an increase in feedback-dominance in the left hemisphere (Figure 2C). The network node degree globally increased in response to propofol, with the strongest hub being present in the parietal area of the brain in under anesthesia (Figure 2B). The joint, univariate and concatenated LZC decreased under propofol anesthesia (Figure 2D).

During the post-anesthetic state, the anesthetic-induced changes in functional connectivity and complexity were reversed and reapproached the Baseline level (see Supplementary Figure 3). Interestingly, despite feedforward-dominant functional connectivity, the network hub remained in posterior regions.

### Case 3

A 28-year-old female was in a chronic DOC following anoxic brain injury after strangulation. Data acquisition was conducted 18 months post-injury. At the day of recording, the patient was classified to be in UWS (CRS-*R* = 6) (Table 1). Three months post-EEG, the patient’s state was unchanged. Due to the patient’s head position during recording, 24 channels in the left temporal and occipital region were removed in addition to non-brain electrodes prior to analysis (see Supplementary Figure 1).

**FIGURE 3:**
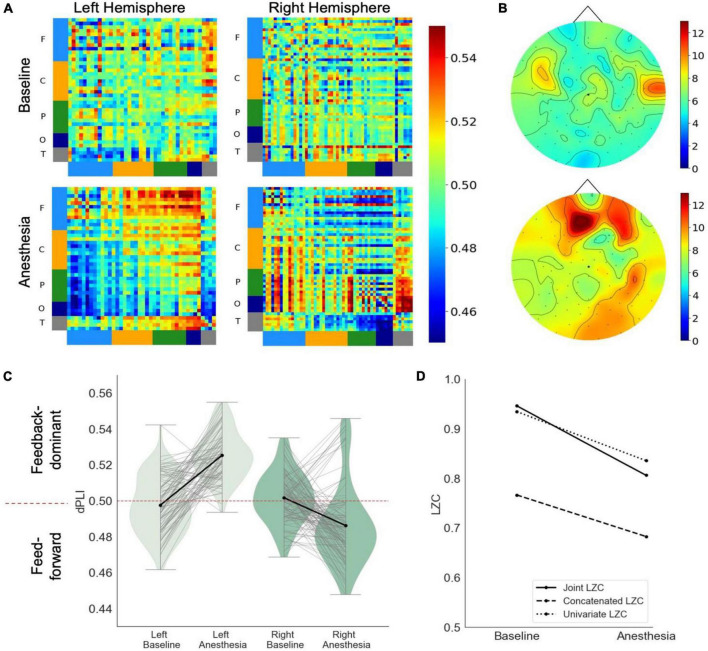
Case 3 **(A)** directed functional connectivity (dPLI) within the right and left hemisphere during baseline and anesthesia. Electrodes are ordered per region: F, frontal; C, central; P, parietal; T, temporal; O, occipital **(B)** network hubs during baseline and anesthesia. **(C)** Directed functional connectivity (dPLI) between frontal and parietal regions within the left and right hemisphere. Light gray lines represent individual electrodes, black lines represent the mean. **(D)** Three types of Lempel-Ziv complexity and its propofol-induced change.

In response to propofol anesthesia, this participant exhibited a strong increase in global feedback-dominant connectivity in the left, but not the right hemisphere (Figure 3A). Focusing solely on connections between frontal and parietal regions revealed an increase in feedback-dominance in the left hemisphere, contrasted by an inhibition of feedback-dominance in the right hemisphere (Figure 3C). The network node degree globally increased in response to propofol, forming an anterior network hub under anesthesia (Figure 3B). The joint, univariate and concatenated LZC decreased under propofol anesthesia (Figure 3D). During the post-anesthetic state, the anesthetic-induced changes in functional connectivity and network hubs were reversed and reapproached the Baseline level (see Supplementary Figure 4). The reduced signal complexity did not revert to Baseline level during the post-anesthetic state.

## Discussion

Many attempts have been made to link neural mechanisms to different states of consciousness. Studies across multiple species and multiple laboratories have accumulated evidence that the disruption of large-scale network organization is associated with disrupted consciousness during sleep (Spoormaker et al., 2010), general anesthesia induced by divergent drugs (Boveroux et al., 2010; Jordan et al., 2013; Lee H. et al., 2013; Bonhomme et al., 2016; Vlisides et al., 2017) and pathological states of consciousness (Vanhaudenhuyse et al., 2010; Ovadia-Caro et al., 2012; Crone et al., 2014; Di Perri et al., 2016). These results have been used to support the major theories of consciousness that are underpinned by the requirement for functional, directed, or effective cortical connectivity in the conscious state (Baars et al., 2013; Oizumi et al., 2014; Mashour et al., 2020). In this paper, we present counterexamples that may challenge the candidacy of the proposed biomarkers of consciousness that have emerged from these theoretical and empirical studies. Using EEG recorded from three patients in DOC, we show that exposure to propofol anesthesia can result in (1) increased frontoparietal feedback-dominant connectivity; (2) posterior network hubs and; (3) increased spatiotemporal complexity. While a previous study has demonstrated that wakefulness can be dissociated from cortical connectivity in rats (Pal et al., 2020), this study demonstrates such dissociation in a case series of three humans. Although the humans in this study were in a DOC, which is associated with fluctuating and unknown levels of consciousness, this condition does not explain the paradoxical markers of consciousness that appear when these patients are exposed to anesthesia. Regardless of the presence or absence of covert consciousness at baseline, exposure to propofol should theoretically strengthen markers of unconsciousness or leave the brain network uninfluenced. The case examples presented in this paper challenges the role of these EEG measures of brain connectivity and complexity in distinguishing states of human consciousness and unconsciousness, and in the clinical monitoring of consciousness (Rosanova et al., 2012; Jordan et al., 2013; Lee U. et al., 2013). Moreover, our findings contribute to growing evidence emerging from conditions such as Angelman syndrome, schizophrenia and non-convulsive epilepsy that present a paradoxical dissociation between a neural marker (e.g., high amplitude delta oscillations) and its putative associated conscious state (see Frohlich et al., 2021 for a full review). This body of work defines a set of boundary conditions must be considered in the quest to refine existing biomarkers of conscious state and select amongst theories of consciousness.

To date, the disruption of feedback-dominant connectivity (i.e., directed connectivity that is most prominent from anterior to posterior regions of the brain) has been one of the top candidate biomarkers of anesthetic-induced unconsciousness (Lee et al., 2009; Nallasamy and Tsao, 2011 as a review). Neutralization or even reversal of feedback-dominant connectivity in the frontoparietal network has been observed in states of unconsciousness induced by various anesthetic agents, including ketamine, propofol and sevoflurane (Ku et al., 2011; Lee U. et al., 2013; Blain-Moraes et al., 2014; Ranft et al., 2016; Lee et al., 2017). In contrast to the expected biomarker of anesthetic-induced unconsciousness, the three case examples presented in this study showed increased frontoparietal feedback connectivity in one (case 2 and 3) or both (case 1) hemispheres during exposure to propofol. It is important to note that these increases were observed using the dPLI to characterize the directed functional connectivity. This distinguishes our results from those of studies that have used linear measures of directional connectivity such as Granger causality, which have also consistently shown opposite results (Nicolaou et al., 2012; Maksimow et al., 2014). Modeling studies have shown that the choice of techniques and variables in these analyses critically affect the net directionality, and that discrepancies arise as intermediate network coupling strengths (Moon et al., 2015). The paradoxical increase in net frontoparietal feedback connectivity observed in our case studies is not a result of disparate connectivity metrics, but instead a reflection of an unexpected change in the underlying network.

The phenomenon of reduced cortical connectivity may be related to the structural and functional architecture of the brain network, which are built around highly connected nodes called hubs (Bassett and Bullmore, 2017). As hubs play a key role in optimizing the computational properties of a network, the disruption of these structures may explain the breakdown in communication that results in unconsciousness. Network hubs are concentrated in the “posterior hot zone” in conscious states; shifts away from this topography may be a marker of the unconscious state (Koch et al., 2016 as a review; Ihalainen et al., 2021). For example, hubs in an EEG network were dominant in anterior brain regions during exposure to propofol anesthesia (Lee H. et al., 2013), and hubs in an fMRI network were radically reorganized in coma patients (Achard et al., 2012). Case example 1 and 2 in this manuscript present an interesting counterexample: upon exposure to propofol anesthesia, the patients’ network hubs became concentrated in posterior brain regions. While network hubs are often treated as a distinct analysis from directed functional connectivity, these approaches are entwined. Empirical studies have demonstrated that the anteriorization of network hubs generally accompanies the reduction of feedback-dominant connectivity (Lee H. et al., 2013; Blain-Moraes et al., 2016), and modeling studies have shown that the direction of information flow in a network can be predicted based on the underlying topology (Moon et al., 2015). The other case example presented in this manuscript demonstrate that the network hub location and the direction of information flow can in fact be decoupled. In case examples 3, exposure to propofol anesthesia results in an increased feedback-dominant connectivity and anterior-dominant network hubs. These empirical examples of decoupling challenge the predictive relationship between network topology and information flow. Specifically, computational models have demonstrated that network hubs are the target of information flow, with peripheral nodes as the sources (Moon et al., 2015). To further explore the decoupling observed in this case example, we conducted a *post-hoc* analysis on the directionality of network hubs (Supplementary Methods A). The results were unexpected: in case example 3, network hubs were both source and target, with a lateral direction of information flow (Supplementary Figure 5). Thus, these counterexamples may prompt not only a reconsideration of network hub topology as a biomarker of consciousness, but also of the fundamental underlying relationship between network topology and directional connectivity in the injured brain.

The dynamic repertoire and the complexity of the brain network have also been related to consciousness (Tononi and Edelman, 1998; Guevara Erra et al., 2016). Of the many measures of neurophysiological complexity, one of the most popular is the LZC, which is grounded in information theory and uses compression to quantify the information content (i.e., amount of non-redundant information) of spontaneous brain activity. The LZC of the brain network is consistently reduced in unconscious states, including anesthesia, sleep and DOC (Sitt et al., 2014; Schartner et al., 2017; Wang et al., 2017). In a recent study in children with Angelman syndrome, the LZC of participants’ EEG tracked the presence and absence of volitional behavioral and wakefulness despite the presence of large delta oscillations in both wakefulness and NREM sleep (Frohlich et al., 2020). The reduction in complexity during anesthesia specifically has been shown in rats (Hudetz et al., 2016) and humans using propofol, isoflurane and sevoflurane (Zhang et al., 2001; Schartner et al., 2015; Toker et al., 2022). These observations have given rise to the hypothesis that a disruption in the dynamic repertoire of the brain is associated with reduced levels of consciousness, and may play a causal role in its suppression. The cases presented in this paper provide counterexamples to this emerging hypothesis: case example 1 demonstrated increased spatiotemporal signal complexity during exposure to propofol.

We consider several potential explanations for the observed paradoxical appearance of putative markers of consciousness under exposure to propofol. First, it is possible that cortical connectivity, network topology and complexity are not biomarkers of the conscious state. This argument has been supported by a previous study in rats, which also showed a dissociation between levels of consciousness and similar biomarkers (Pal et al., 2020). However, this explanation is challenged by the absence of drug-specific neural markers of propofol-induced unconsciousness in our participants (Purdon et al., 2013), which we would expect to see in response to the presence of the anesthetic, regardless of level of consciousness. Second, it is possible that the proposed biomarkers are only associated with levels of consciousness in brains with sufficient structural integrity. In cases of patients in DOC with severe brain injuries and significant structural damage, it is possible that the underlying physical network architecture is unable to give rise to the expected markers of consciousness. Functional connectivity exhibits an increased resemblance to structural network organization in unconscious states (Barttfeld et al., 2015); thus, disrupted structural networks may explain the paradoxical network reorganization under propofol anesthesia. While this is not the case in all DOC (Blain-Moraes et al., 2016; Duclos et al., 2022), the minimum necessary structure that supports expected markers of consciousness remains an open question. Third, we consider the possibility that cortical connectivity, network topology and complexity are true biomarkers of the conscious state, indicating that the level of consciousness of the case examples presented in this paper improved upon exposure to anesthesia. This explanation is supported by previous studies that have shown that a sedative GABAergic drug – zolpidem – can increase arousal in patients with DOC (Du et al., 2014; Machado et al., 2014; Noormandi et al., 2017; Sripad et al., 2020). These observations can be reconciled using the hypothesis that consciousness relies on brain networks which are tuned to criticality, a narrow window between stability and chaos; disorder and order (Tagliazucchi, 2017; Popiel et al., 2020; Toker et al., 2022). In this framework, deviations from criticality to either the stable or the chaotic side result in unconsciousness and loss of advantageous network properties such as signal complexity (Tagliazucchi, 2017; Popiel et al., 2020; Toker et al., 2022). Although most patients in a DOC are expected to deviate from criticality on the side of increased chaoticity (i.e., closer to the anesthetized state), Toker et al. (2022) reported one patient who surprisingly increased chaoticity after recovery of consciousness. This raises the possibility that DOC are a heterogenous set of conditions, which can be either too chaotic (i.e., comparable to the state of propofol-anesthesia), or too stable (i.e., comparable to the state of epileptic seizures). The three case studies present in this paper potentially represent a less-typical DOC phenotype, deviating from criticality on the side of too much stability. Under these circumstances, the biomarkers of consciousness remain associated with conscious state, as exposure to propofol would bring these patients closer to a critical state and thus increase their level of consciousness. Fourth, it is possible that observed changes in cortical connectivity, network topology and complexity were underpinned by differences in signal quality and noise level, rather than changes of conscious states. However, all three states of EEG data acquisition were performed within a maximal time of 2 h. Impedances were reduced to below 5 kΩ prior to every state’s data collection. Additional evidence that the observed changes were induced by the effect of anesthesia, rather than differences in signal quality is provided by the similarity between the baseline and post-anesthetic state (see Supplementary Figures 2–4).

The results of this case series analysis must be interpreted in light of several limitations. First, we analyzed cortical connectivity and network hubs only in the alpha frequency band (8–13 Hz), which has previously been implicated in studies of consciousness (Lee H. et al., 2013; Blain-Moraes et al., 2014, 2017; Kallionpää et al., 2020). It is possible that the observed changes in connectivity and network topology are epiphenomenal to known shifts in alpha power resulting from propofol anesthesia (Purdon et al., 2013). However, in this study only case 3 demonstrated a strong increase in alpha power in response to Propofol (see Supplementary Figure 1). Additionally, we calculated the correlation between the propofol-induced increase in alpha power and the change in node degree and demonstrated that there was no significant correlation (Supplementary Figure 6). The absence of an alpha peak in case 1 and 2 leads to a second limitation of this study: the analysis in this frequency band despite the absence of oscillatory peaks. In human EEG, the oscillatory component (i.e., peaks in the power spectral density) always co-exists with a non-oscillatory, aperiodic component (i.e., exponential decay of power over frequency) (Donoghue et al., 2020). Interpreting the aperiodic component erroneously as oscillation may lead to several methodological problems and incomplete representation of underlying neurophysiological processes (Donoghue et al., 2021). Indeed, the aperiodic component of the human EEG has been shown to be sensitive to effects of anesthesia (Colombo et al., 2019; Lendner et al., 2020). Further research is needed to investigate dependence of the investigated markers of consciousness on changes in the aperiodic component.

Third, this case series only includes female individuals in DOC. Whereas sex and gender is known to influence theoretical and practical pharmacokinetics of propofol (Vuyk et al., 2001; Kodaka et al., 2006), no work has been done on gender difference in biomarkers of propofol-induced unconsciousness. Fourth, the anesthetized state in this study was defined by a target effect size concentration of 2 μg/ml. Thus, it is possible that participants were in a state of deep sedation, rather than fully anesthetized. However, even in a state of deep sedation, the paradox of the brain response to propofol in this case series remains and continues to challenge candidate biomarkers of conscious level. Fifth, the post-anesthetic state was recorded after the concentration of propofol has reached 0.5 μg/ml. Despite the low concentration of Propofol at this time we cannot exclude a remaining effect on individual’s brain function.

Cumulatively, the paradoxical reconfiguration of brain networks of DOC patients undergoing exposure to anesthesia prompts a re-evaluation of two of the top candidate biomarkers of consciousness level: connectivity and complexity. Accounting for these counterexamples may be a source of insight and guidance toward the identification of generalizable biomarkers that underpin human consciousness.

## Data availability statement

Data will be made available upon reasonable request. Requests to access these datasets should be directed to SB-M, stefanie.blain-moraes@mcgill.ca.

## Ethics statement

The studies involving human participants were reviewed and approved by the McGill University Health Center Research Ethics Board (15-996-MP-CUSM). Written informed consent was not provided because participants were unresponsive at the time of data collection. Written informed consent was provided by their legal representative in accordance with the Declaration of Helsinki.

## Author contributions

CM, CD, and SB-M designed the research. CM performed the analysis. CM and SB-M wrote the manuscript. CD and SB-M provided feedback. All authors contributed to the article and approved the submitted version.
