# Supplementary material for: Paradoxical markers of conscious levels: Effects of propofol on patients in disorders of consciousness
Source: Front Hum Neurosci. 2022 Oct 6;16:992649. doi: 10.3389/fnhum.2022.992649 (PMC9584648; doi:10.3389/fnhum.2022.992649)
Supplement: Supplementary file 1 [file Data_Sheet_1.pdf]

Supplementary Material

Paradoxical markers of conscious levels: Effects of propofol on patients in disorders of consciousness

Charlotte Maschke, Catherine Duclos, Stefanie Blain-Moraes

- **Supplementary Methods**

Directed network hubs were estimated based on the electrode-wise node degree (i.e. summed connection from one electrode to all other electrodes) of the time-averaged zero-centered dPLI matrix. Negative values represent the source of information flow and positive values indicate the target of information flow. The power spectral density was estimated using the Multitaper approach for every electrode and epoch individually. Alpha power was defined by the average power in the 8-13 Hz range. The relation between alpha power and node degree was estimated using Pearson correlation.

- **Supplementary Figures and Tables**

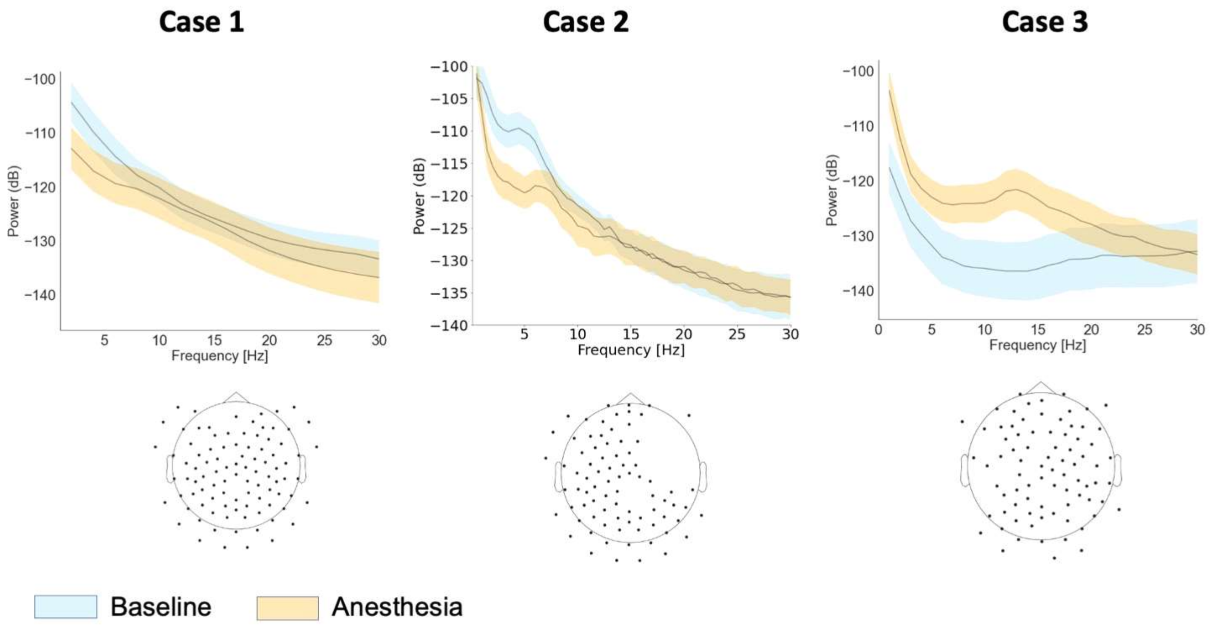

Supplementary Figure 1. Power spectral density and electrode distribution for Case 1, 2 and 3 during Baseline and Anesthesia. Electrode distribution corresponds to all three recorded states.

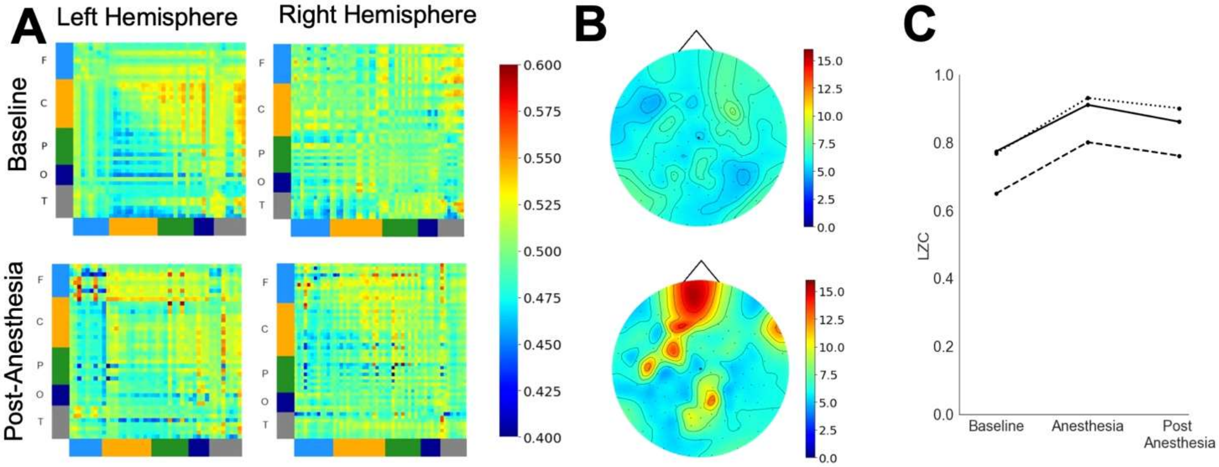

Supplementary Figure 2. Case 1 (A) directed functional connectivity (dPLI) within the right and left hemisphere during baseline and post-anesthesia. Electrodes are ordered per region: F: Frontal, C: Central, P: Parietal, T: Temporal and O: Occipital (B) Network hubs during baseline and post-anesthesia. (C) three types of Lempel-Ziv complexity and change during and after propofol.

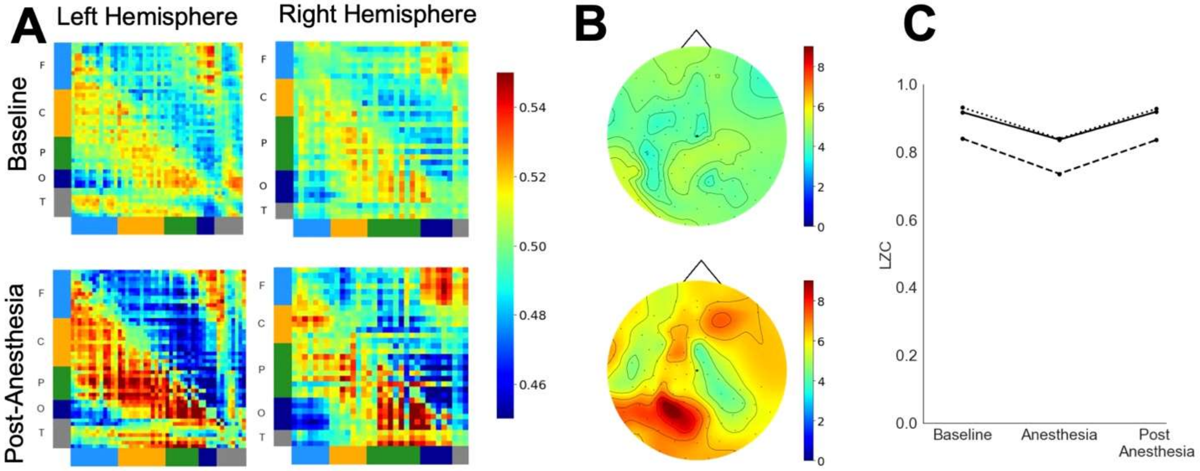

Supplementary Figure 3. Case 2 (A) directed functional connectivity (dPLI) within the right and left hemisphere during baseline and post-anesthesia. Electrodes are ordered per region: F: Frontal, C: Central, P: Parietal, T: Temporal and O: Occipital (B) Network hubs during baseline and post-anesthesia. (C) three types of Lempel-Ziv complexity and change during and after propofol.

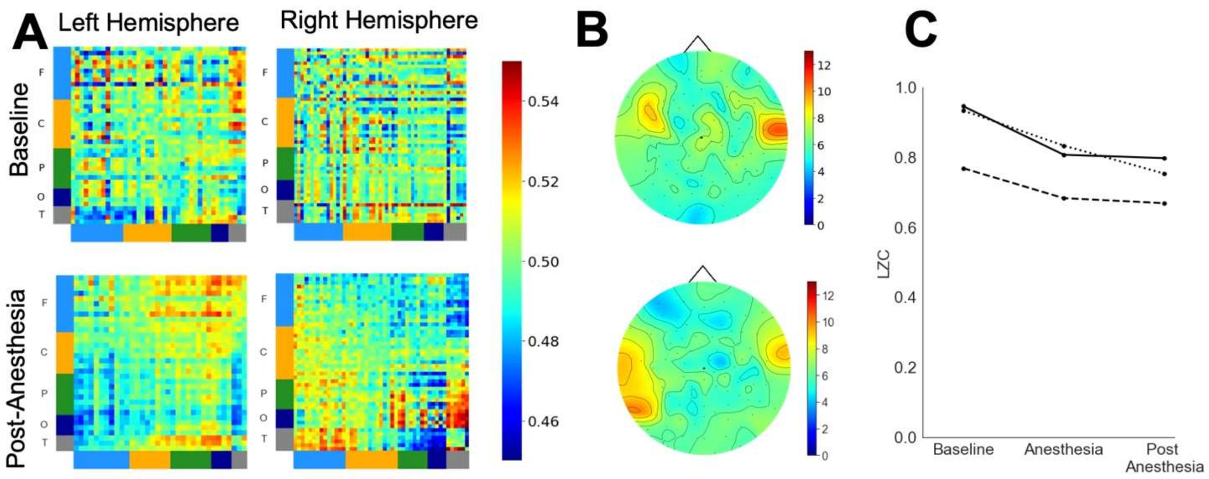

Supplementary Figure 4. Case 3 (A) directed functional connectivity (dPLI) within the right and left hemisphere during baseline and post-anesthesia. Electrodes are ordered per region: F: Frontal, C: Central, P: Parietal, T: Temporal and O: Occipital (B) Network hubs during baseline and post-anesthesia. (C) three types of Lempel-Ziv complexity and change during and after propofol.

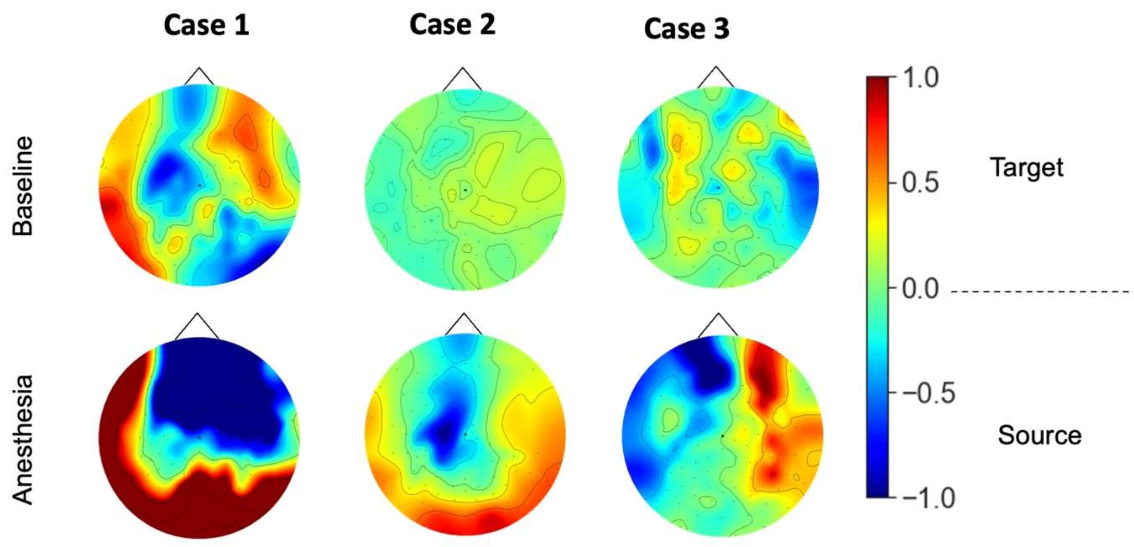

Supplementary Figure 5. Directed Network hub of the three presented cases during baseline condition and exposure to propofol anesthesia. Blue areas represent sources of information, red areas target of information flow.

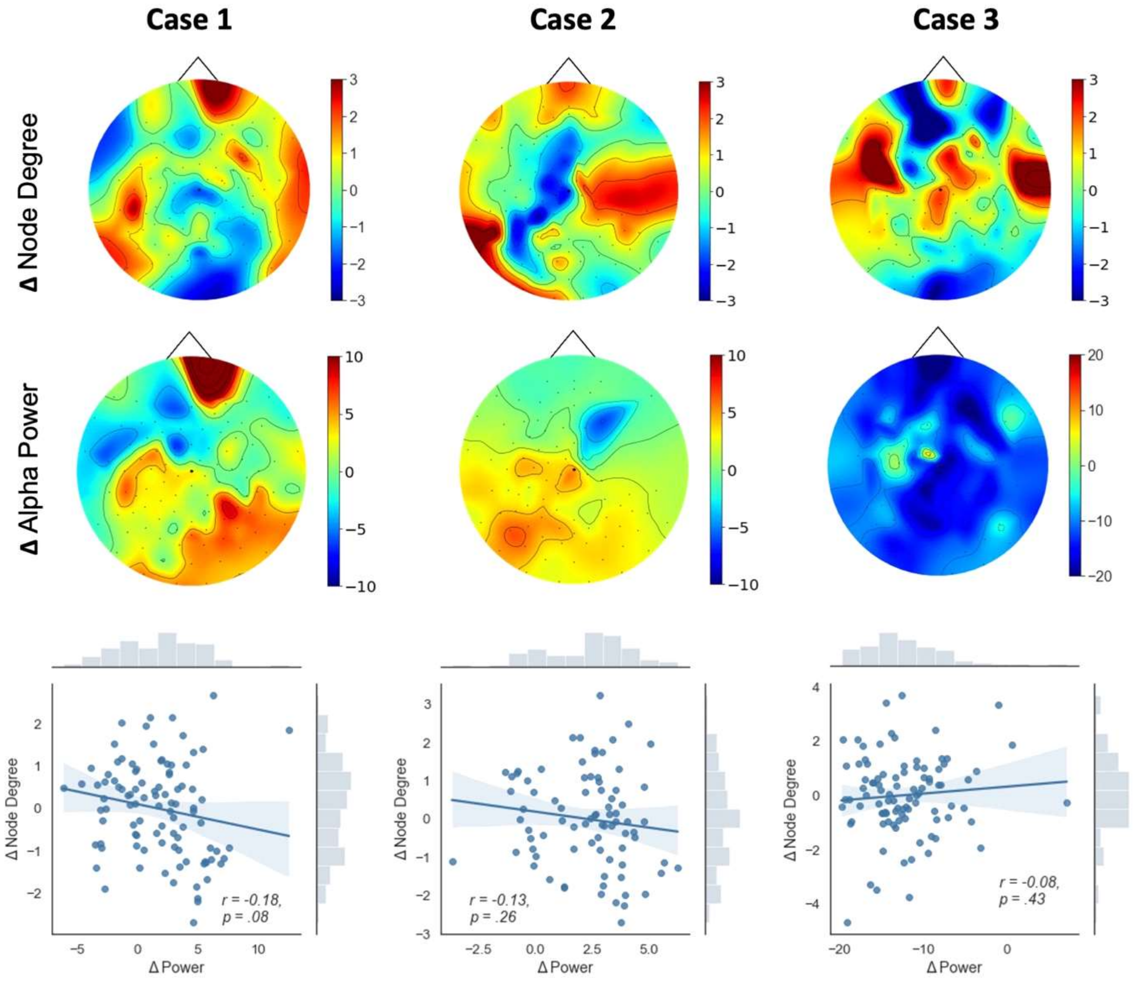

Supplementary Figure 6. Dissociation between the propofol-induced increase in alpha power and the change in node degree in the three clinical cases. There was no significant correlation Δ Node Degree (i.e. Degree Baseline - Degree Anesthesia) and Δ Alpha power (i.e. Power Baseline - Power Anesthesia).
